# Docosahexaenoic acid suppresses breast cancer cell metastasis by targeting matrix-metalloproteinases

**DOI:** 10.18632/oncotarget.10266

**Published:** 2016-06-23

**Authors:** Eun-Jin Yun, Kyung-Sub Song, Soyeon Shin, Soyeon Kim, Jun-Young Heo, Gi-Ryang Kweon, Tong Wu, Jong-Il Park, Kyu Lim

**Affiliations:** ^1^ Department of Biochemistry, College of Medicine, Chungnam National University, Daejon 301-747, Republic of Korea; ^2^ Infection Signaling Network Research Center, Chungnam National University, Daejon 301-747, Republic of Korea; ^3^ Cancer Research Institute, Chungnam National University, Daejon 301-747, Republic of Korea; ^4^ Department of Urology, University of Texas Southwestern Medical Center, Dallas, TX 75390, USA; ^5^ Department of Pathology and Laboratory Medicine, Tulane University School of Medicine, New Orleans, LA 70112, USA

**Keywords:** DHA, omega-3 PUFA, MMP, breast cancer

## Abstract

Breast cancer is one of the most prevalent cancers in women, and nearly half of breast cancer patients develop distant metastatic disease after therapy. Despite the significant advances that have been achieved in understanding breast cancer metastasis in the past decades, metastatic cancer is still hard to cure. Here, we demonstrated an anti-cancer mechanism of docosahexaenoic acid (DHA) that suppressed lung metastasis in breast cancer. DHA could inhibit proliferation and invasion of breast cancer cells *in vitro*, and this was mainly through blocking Cox-2-PGE_2_-NF-κB-MMPs cascades. DHA treatment significantly decreased Cox-2 and NF-κB expression as well as nuclear translocation of NF-κB in MDA-MB-231 cells. In addition, DHA also reduced NF-κB binding to DNA which may lead to inactivation of MMPs. Moreover, *in vivo* studies using Fat-1 transgenic mice showed remarkable decrease of tumor growth and metastasis to EO771 cells to lung in DHA-rich environment. In conclusion, DHA attenuated breast cancer progression and lung metastasis in part through suppressing MMPs, and these findings suggest chemoprevention and potential therapeutic strategy to overcome malignant breast cancer.

## INTRODUCTION

Despite considerable diagnostic and therapeutic advances, breast cancer is still the most common malignancy found in women [1]. Localized early-stage breast tumors are managed by surgery, radiation and estrogen depleting therapy. Excessive estrogen exposure promotes breast carcinogenesis by increasing tumor cell proliferation and suppressing DNA repair mechanisms and drugs inhibiting estrogen signaling have been used successfully for the treatment of early and advanced stage of breast cancer. The mortality of breast cancers has decreased and the five-year overall survival rate has increased to 90% since 1990, however, once breast cancer metastasis occurs, they rarely respond to treatment [2]. Approximately 40% of patients with localized breast cancer have micrometastatic disease that is difficult to detect at the time of diagnosis and treatment, and result in disease recurrence and death at several years after diagnosis [3-5]. Metastasis to the lung, spine, ribs, pelvis, and proximal long bones are frequently seen in pathological lesions in advanced breast cancer [1].

Among dietary factors, ω6-polyunsaturated fatty acids (ω6-PUFAs) and saturated fatty acids which are the principal confounding factors of breast cancer [6]. Dietary lipid rich in ω6-PUFA containing arachidonic acid (AA) were found to stimulate the growth and metastasis of human breast cancer cells through increased synthesis of cyclooxygenase (Cox) and lipoxygenase (Lox) catalyzed products [7, 8]. In contrast, there are growing epidemiological, clinical and experimental evidences have suggested a protective effect of ω3-PUFAs on breast cancer [9-12]. Docosahexaenoic acid (DHA) and eicosapentaenoic acid (EPA) are typical ω3-PUFAs, which have been shown to affect: hormone (eicosanoid) production [13], formation of potent lipid peroxidation products [14], the conformation and hence activity of specific enzymes [15], transcription events [16], and membrane structure and function [17]. Recently, we also published several papers on novel mechanisms of DHA including induction of autophagy [18] and activation of ubiquitin/proteasome system in cancer cells [19]. In this study, we investigated the anti-proliferative and anti-invasive effects of DHA in human breast cancer cell line MDA-MB-231, and the results demonstrated that DHA significantly inhibited various oncogenic pathways including β-catein, NF-kB and Cox2. In addition, we found that DHA inhibited invasiveness of the cells by suppressing MMP-2 and MMP-9 transcription through blocking Cox2-PGE_2_-NF-kB cascade. Importantly, we confirmed inhibitory effects of DHA on *in vivo* tumor growth and metastasis using Fat-1 transgenic mice which maintained high-levels of ω3-PUFAs including DHA in their tissue. In conclusion, this study provides the first direct evidence that DHA suppress breast cancer lung metastasis in preclinical *in vivo* tumor models as well as invasion and migration in *in vitro* tumor cells. These findings provide important preclinical evidence and molecular insight for the use of DHA in the chemoprevention as well as potential therapeutic regimen to overcome highly invasive and aggressive breast cancer.

## RESULTS

### DHA leads to apoptotic cell death in breast cancer cell lines

Several studies have suggested anti-proliferative and cytotoxic effects of DHA [11, 20, 21]. To confirm this, we first performed MTT cell proliferation assay with different concentration of DHA, EPA and AA. Indeed, both ω3-PUFAs, DHA and EPA decreased the cell growth, and DHA exhibited more significant effect shown 80% growth inhibition at 50 μM concentration (Supplementary Figure 1A and 1B). On the other hand, ω6-PUFA, AA didn't decrease the cell growth in MDA-MB-231, even slightly increased the cell growth in T47D cells (Supplementary Figure 1A). In addition, DHA treatment significantly increased the subG1 cell population rather than inducing G1, S or G2-M phase arrest (Supplementary Figure 2A). To further identify the cytotoxic effect of DHA, we examined the effects of DHA on markers of apoptosis including PARP cleavage, caspase-3 activity. The results showed that DHA triggered PARP cleavage in a dose-dependent manner, and caspase-3 activity was also significantly increased after treatment of 10-25 μM DHA (Supplementary Figure 2B and 2C) suggesting that accumulation of subG1 phase and induction of apoptotic signaling might be the key mechanisms for growth inhibition effect of DHA.

**Figure 1:**
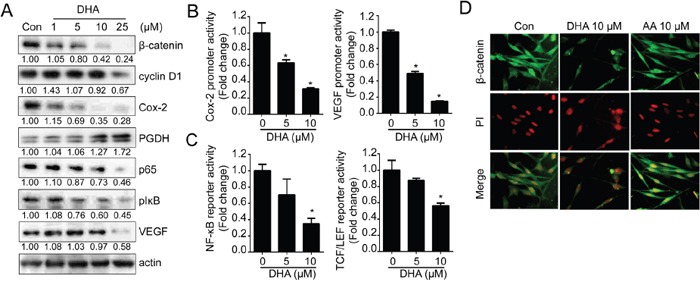
DHA suppresses various oncogenic signaling pathways **A.** MDA-MB-231 cells were treated with various concentration of DHA for 24 hrs and the expression of β-catenin, cyclin D1, Cox-2, PGDH, p65, pIκB, and VEGF were analyzed by Western blot analysis. The densitometric ratio to actin band intensity for each sample was normalized to the control and shown below the blot. **B.** MDA-MB-231 cells were transfected with luciferase reporter containing Cox-2 and VEGF promoter and treated with DHA for 24 hrs. Then, the cells were lysed and the luciferase activity was measured with the dual luciferase assay. *, *p* <0.01 compared with control, Student's *t* test, significantly different from the control. **C.** MDA-MB-231 cells were transfected with luciferase reporter containing TCF/LEF or NF-κB binding site and treated with DHA for 24 hrs. **D.** MDA-MB-231 cells were treated with DHA (10 μM) or AA (10 μM) for 24 hrs, and cells were immunostained with anti-β-catenin antibody.

Furthermore, the expressions of various oncogenic signal molecules including β-catenin, Cox-2 and NF-κB were decreased by DHA treatment (Figure 1A). As shown in Figure 1B, DHA treatment decreased not only protein expression but also promoter activities as well. The luciferase reporter containing Cox-2 and VEGF promoter region significantly suppressed their activity by DHA in a dose dependent manner. In addition, DHA decreased the activity of reporter containing NF-κB and TCF/LEF binding region (Figure 1C). Furthermore, DHA induced the depletion of β-catenin in nucleus suggesting that DHA inhibited translocation of β-catenin and blocked its transcriptional activity (Figure 1D).

### DHA reduces *in vitro* cell invasion and motility by suppressing MMP expression and activity

**Figure 2:**
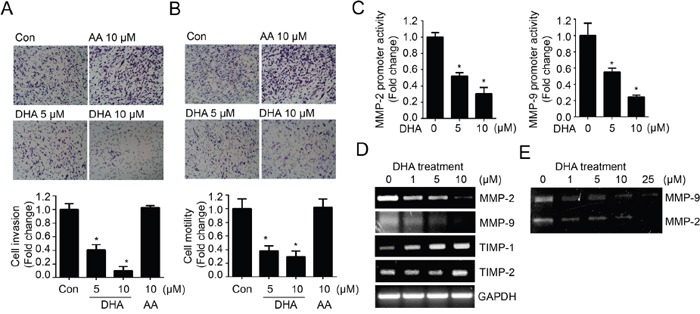
DHA treatment reduces *in vitro* cell invasion and motility by modulating MMP expression and activity **A.** MDA-MB-231 cells loaded onto Matrigel-coated upper chamber transwell were treated with DHA (5, 10 μM) or AA (10 μM) 24 hrs. Then the numbers of invasive cells were counted in 10 random fields and normalized by Con cells as 1. **B.** MDA-MB-231 cells were loaded onto upper chamber transwell without Matrigel, and treated with DHA or AA for 24 hrs. The filtrated cells were stained and quantified. **C.** MDA-MB-231 cells transfected with MMP-2 or MMP-9 promoter were treated with increased dose of DHA (0, 5, 10 μM) for 24 hrs. Then, the cells were lysed and the luciferase activity was measured with the dual luciferase assay. **D.** MDA-MB-231 cells were treated with increased dose of DHA (0-10 μM) for 24 hrs and MMP-2, MMP-9 and TIMPs mRNA levels were analyzed by RT-PCR. **E.** MDA-MB-231 cells were treated with increased dose of DHA (0-25 μM) for 24 hrs, and condition media were collected and MMP activity was analyzed using gelatin zymography for secreted enzymatic activity.

Next, we investigated whether DHA can suppress the metastasis of breast cancer cell. Metastasis consists of a complex cascade of events involving cell adhesion, invasion and motility [22]. To determine the effect of DHA on invasiveness and motility, we performed *in vitro* invasion and motility assay with MDA-MB-231 cells. Noticeably, DHA showed inhibitory effect on cell invasion through the Matrigel chamber (Figure 2A); DHA decreased the invasion by approximately 10-fold. Consistently, the treatment of MDA-MB-231 cells with DHA also led to a dose-dependent decrease in cell motility by Boyden chamber assay as well (Figure 2B). Cell motility is a critical process of invasion allowing primary tumors to metastasize, and invasion is principally stimulated by the gelatinase matrix-metalloproteinases (MMPs), and especially MMP-2 and MMP-9 were found to elevate in breast cancer and associated with disease progression [23, 24]. To determine the relationship between the MMPs and reduced-invasiveness by DHA, we next examined the expression of MMP-2 and MMP-9. As like other oncogenic molecules, the treatment of DHA decreased the MMP-2 and MMP-9 promoter activity (Figure 2C) and followed by their mRNA expression (Figure 2D). On the other hands, the expressions of MMPs inhibitor TIMP-1 and TIMP-2 were increased by DHA (Figure 2D). To further test whether reduced expression of MMPs leads to their activity, gelatin zymography was performed and the result showed the activities of both MMP-2 and MMP-9 were decreased by the treatment of DHA (Figure 2E). Together, these results suggest that DHA decreases *in vitro* invasion and motility by suppressing MMP-2 and MMP-9 expression as well as their activity.

### DHA suppresses MMP-2 and MMP-9 by antagonizing PGE_2_

**Figure 3:**
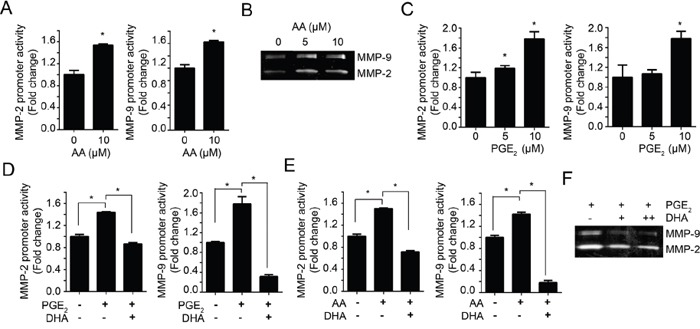
DHA antagonizes the effect of PGE_2_ **A.** MDA-MB-231 cells transfected with MMP-2 or MMP-9 promoter were treated with 10 μM AA for 24 hrs. Then, the cells were lysed and the luciferase activity was measured with the dual luciferase assay. **B.** MDA-MB-231 cells were treated with increased dose of AA (0, 5, 10 μM) in serum-free media for 24 hrs. Then the condition media were prepared and the activity of MMP-2 and MMP-9 were analyzed using gelatin zymography. **C.** MDA-MB-231 cells were transfected with MMP-2 and MMP-9 promoter, and then treated with PGE_2_ (0, 5, 10 μM) for 12 hrs and luciferase activity was measured with the dual luciferase assay. **D-E.** MDA-MB-231 cells transfected with MMP-2 or MMP-9 promoter were pretreated with DHA (10 μM) for 2 hrs, and then PGE_2_ (10 μM) (D) or AA (10 μM) (E) were added, respectively. After incubation for 12 hrs, the luciferase activity was measured with dual luciferase assay. **F.** MDA-MB-231 cells were treated with PGE_2_ (10 μM) in presence or absence of DHA (10 μM), and the activity of MMP-2 and MMP-9 promoter were analyzed by gelatin zymography.

In contrast with DHA, AA treatment increased both the promoter activities and enzyme activities of MMP-2 and MMP-9 (Figure 3A and 3B). AA is the primary precursor for prostaglandin E_2_ (PGE_2_) (Supplementary Figure 3A) and these oxygenated metabolites can promote tumor initiation and progression by enhancing cell proliferation, angiogenesis, cell migration and invasion, while inhibiting apoptosis [25, 26]. To investigate whether AA-induced MMP promoter activity was caused by increased PGE_2_, we examined the effect of exogenous PGE_2_ on MMP-2 and MMP-9 promoter activity. As shown in Figure 3C, the promoter activity of MMP-2 and MMP-9 was increased by PGE_2_ in a dose-dependent manner. Interestingly, we observed that AA-induced or PGE_2_-induced promoter activities were significantly blocked by pretreatment of 10 μM DHA (Figure 3D, 3E). These results indicate that DHA inhibit AA-induced PGE2 production as well as increase the exogenous PGE_2_ degradation. As shown in Figure 3F, we also found that DHA inhibits PGE_2_-induced MMPs activities. Cox-2 is important enzyme that produces PGE_2_ from AA [27, 28]. Therefore, we next examined effects of DHA on Cox-2-induced MMP promoter activity. As shown in Supplementary Figure 3B, pretreatment of DHA significantly blocked the Cox-2-induced MMP promoter activity. These results indicate that DHA suppress MMPs expression and activity by inhibiting Cox-2 and inhibiting PGE_2_ production by compete with AA.

### DHA blocks NF-κB signaling pathway by inhibiting IKK activity

**Figure 4:**
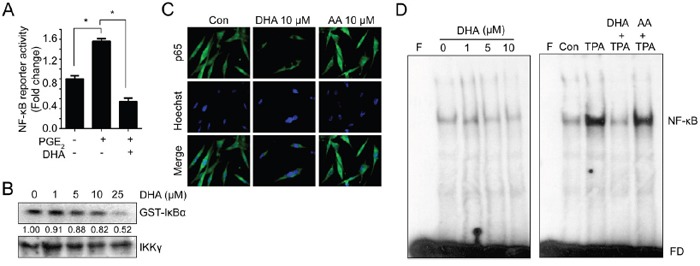
DHA blocks NF-κB signaling which can bind to MMP promoter **A.** MDA-MB-231 cells transfected with reporter construct containing NF-κB binding site were pretreated with DHA (10 μM) for 2 hrs, and then PGE_2_ (10 μM) were added. After 12 hrs, the luciferase activity was measured with dual luciferase assay. **B.** Cells were treated with increased dose of DHA (0-25 μM) for 24 hrs, and whole cell lysates were immunoprecipitated with anti-IKKγ. Then immune complex kinase assay was performed with [γ-^32^P]ATP and GST-IκB-α as an exogenous substrate. GST-IκB-α phosphorylation was assessed by SDS-PAGE and autoradiography. The densitometry ratio to IKKγ band intensity for each sample was normalized to the control and shown below the blot. **C.** MDA-MB-231 cells were treated with DHA (10 μM) or AA (10 μM) for 24 hrs, and cells were immunostained with p65 antibody. **D.** MDA-MB-231 cells were treated with DHA for 24 hrs. To induce binding, TPA was pretreated for 4 hrs and then DHA was added. And the nuclear extracts were incubated with the radiolabeled oligonucleotide containing NF-κB and EMSA was performed. F, blank without nuclear extract; FD, free DNA probe.

NF-κB is a master regulator of gene expression that related with proliferation, inflammation and invasion [29, 30]. Since MMPs promoters commonly contain NF-kB binding site, we examined effects of PGE_2_ on NF-kB activation. As shown Figure 4A, PGE2 induced NF-kB reporter activity and these effects attenuated by pretreatment with DHA. These results suggest that PGE2-induced MMPs promoter activity may be increased by NF-kB pathway. In previous result, DHA inhibits endogenous NF-kB reporter activity in a dose dependent manner. Therefore we further examined whether DHA could directly inhibit NF-kB pathway. The degradation of IκBs stimulated their phosphorylation by IκB kinase (IKK), and it leads to the release of NF-κB which allows its translocation into the nucleus and subsequent activation of a number of target genes [31, 32]. To identify the role of DHA on NF-κB pathway, we assessed the *in vitro* IκB kinase (IKK) assay. As shown in Figure 4B, treatment of DHA decreased IκBα phosphorylation in a dose-dependent manner, suggesting that DHA inhibits IKK activity *in vitro*. In addition, DHA blocked the nuclear translocation of p65 (Figure 4C). Next, we examined the effect of DHA on DNA binding affinity of NF-κB. ^32^P-labeled probe containing the NF-κB motif and nuclear extract from DHA-treated cells were mixed, and the formation of a DNA-protein complex was analyzed by EMSA. The result showed that the NF-κB binding was decreased by DHA in a dose dependent manner (Figure 4D, left panel). To further confirm the decreased binding by DHA, the cells were pretreated with 12-O-tetradecanoylphorbol-13-acetate (TPA) to activate NF-κB. Consistently, NF-κB binding affinity which increased by TPA was abolished by DHA treatment whereas AA couldn't inhibit the binding affinity (Figure 4D, right panel). Together, these results suggest that DHA decreases NF-κB signaling including translocation and binding affinity to promoter region, and these effects may be partially through inhibiting IKK activity.

### ω3-PUFA-rich environment suppresses *in vivo* tumor growth and metastasis

**Figure 5:**
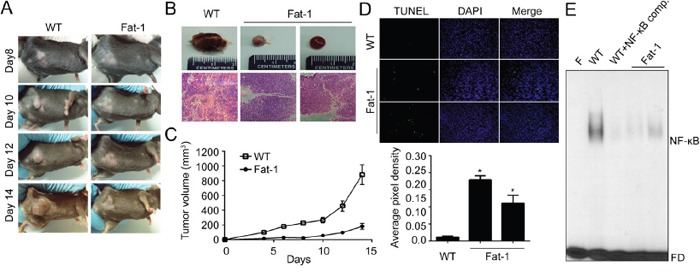
DHA-rich environment retards tumor growth *in vitro* **A-B.** Fat-1 transgenic and C57BL/6 wild-type mice were implanted with murine breast carcinoma cell line EO771 cells. After inoculation, animals were closely monitored for the development of subcutaneous tumor. After two weeks, the animals were sacrificed and xenograft tissues were collected for further experiment. Day of the implantation of the tumor cells was designated day 0. WT, representative xenograft tumor from wild type mouse; Fat-1, two representative xenograft tumors from fat-1 transgenic mice. **C.** The tumor size was measured at indicated time with a caliper. Tumor volume=0.5×(width)^2^×length. **D.** The animals were sacrificed at two weeks after implanted, tumor tissues were fixed with formalin and TUNEL stain was performed. **E.** The nuclear extracts were prepared from mice xenograft tissues. Then the nuclear extracts were incubated with the radiolabeled oligonucleotide containing NF-κB and EMSA was performed. F, blank without nuclear extract; Comp., competitor without isotope labeling; FD, free DNA probe.

**Figure 6:**
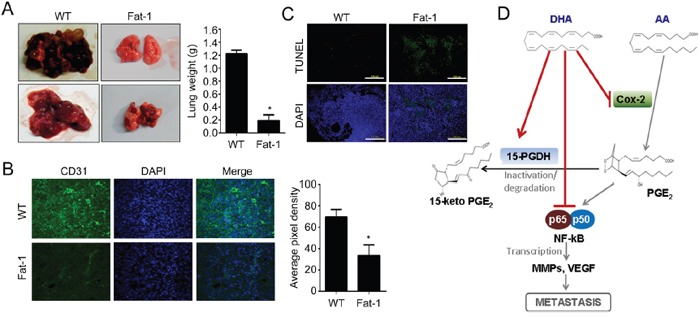
DHA-rich environment prevents lung metastasis **A.** Murine breast carcinoma cell line EO771 cells were injected into tail vein of Fat-1 transgenic and wild-type mice. After 4 weeks, the animals were sacrificed and dissected to check the metastasis to lung. **B.** The xenograft tissue in lung were fixed and stained with endothelial marker CD31 antibody. **C.** The xenograft tissues in lung were fixed with formalin and TUNEL stain was performed. **D.** The mechanism of DHA on suppressing metastasis was illustrated.

To evaluate our finding *in vivo*, we employed Fat-1 transgenic mice as a model of increased ω3-PUFAs tissue status. Fat-1 transgenic mice carry a *Caenorhabditis elegans* ω3 desaturase (*fat-1* gene), leading to the endogenous formation of ω3-PUFAs from ω6-PUFA. Due to this capability, Fat-1 mice have a higher tissue content of ω3-PUFAs compared to wild type (WT) littermates, and increased anti-inflammatory effects [33]. Before implantation of murine breast cancer cell lines (EO771) into mice, effects of DHA on EO771 cells were confirmed *in vitro*. Consistent with the results in human breast cancer model, DHA significantly decreased cell viability and induced apoptotic cell death (Supplementary Figure 4). To compare the tumor incidence in WT vs. Fat-1 mice, EO771 cells were injected subcutaneously into mice. And as shown in Figure 5A-5C, the primary tumor growth from the Fat-1 mice was significantly inhibited compared with that formed from WT. After 14 days, the average tumor volume was decreased by 80% in Fat-1 compared with WT (Figure 5C). Also the increased apoptosis as determined by TUNEL assay was observed in Fat-1 mice (Figure 5D). Consistent with *in vitro* results, NF-κB binding affinity decreased in xenograft tissues derived from Fat-1 mice compare to that from WT mice (Figure 5E). To determine the effect of DHA on the levels of breast cancer cell metastasis to lung, murine breast cancer cell line EO771 was injected into tail vein. As shown in Figure 6A, lung metastasis was significantly suppressed in Fat-1 mice. Additionally, we found that the levels of CD31 which is endothelial marker, was markedly suppressed in tumor tissue derived from Fat-1 mice than WT mice (Figure 6B). Consistently, the xenograft tissues from Fat-1 mice showed higher apoptotic index (Figure 6C). Taken together, this study demonstrated that *in vitro* DHA treatment and *in vivo* DHA-rich environment could prevent breast cancer progression and metastasis by blocking Cox2-PGE2-NFκB pathway (Figure 6D).

## DISCUSSION

Breast cancer is the most common malignant disease in Western women. Particularly, the metastatic spread of primary tumor to distant sites; typically to bone, lung, liver, and brain, accounts for the vast majority of cancer-related deaths [34]. Recently, the rates of metastasis and mortality in breast cancer patients have decreased as a result of improvements in breast cancer treatment and early detection [35, 36]. However, approximately 10-15% of patients with breast cancer still has an aggressive disease and develops distant metastases with 3 years after the initial detection of the primary tumor, and manifestation of metastasis 10 years or more after the initial diagnosis is also not unusual [37]. Patients with breast cancer are therefore at risk of experiencing metastasis for their entire lifetime.

ω6-PUFAs/ω3-PUFAs ratio in diets alter the risk of malignancy, though their exact patho-chemical interactions with the tumor are still obscure. It has been reported that ω6-PUFAs stimulate carcinogenesis, tumor growth and metastasis, whereas ω3-PUFAs have inhibitory effects of them. The proposed mechanisms of ω3-PUFAs on tumor progression is that the formation of potent lipid peroxidation products, the conformation and hence activity of specific enzymes, transcription events, activation of the ubiquitin-proteasome system [19] and membrane structure and functions [38, 39]. It has been also known that the mechanism of ω3-PUFAs-induced cell death relates to the number of skipped dienes and their arrangement involves lipid peroxidation [40].

In this study, we utilized *in vitro* and *in vivo* models to investigate the effect of DHA on growth, invasion, and metastasis in breast cancer. It has been known that DHA and EPA together or alone also inhibit the growth of breast cancer cell line *in vitro* [41, 42]. We also confirmed that the treatment of DHA decreased cell proliferation and induced apoptosis in a dose- and time-dependent manner in MDA-MB-231 human breast cancer cell line. The results demonstrated that DHA had significant cytotoxic effect at least in part through inhibition of Wnt/β-catenin signaling pathways.

In addition to cytotoxic effect, DHA significantly had suppressed invasiveness by suppressing MMP-2 and MMP-9 secretion through inhibition of NF-κB and Cox-2 signaling pathway. The predominant mechanisms of DHA have been thought to be a reduction in pro-inflammatory eicosanoids and an increase in inflammation-resolving derivatives [38, 39]. Particularly, Cox-2 is the major inducible enzyme catalyzing the rate-limiting step in the metabolic pathway that transforms AA into its PGs, and has a crucial role in breast cancer progression [59]. In this study, Cox-2 protein expression and its transcriptional activity were significantly reduced by DHA treatment. In addition, PGE_2_, Cox-2, and AA induced MMP-2 and MMP-9 promoter and enzyme activity whereas DHA treatment suppressed MMP-2 and MMP-9 activity. Generally, Cox-2 is known to up-regulated in most human cancers and PGE_2_ is also produced in large amounts in cancers, and involved in cancer metastasis [43, 44]. Our data showed that pre-treatment of DHA significantly blocked the effect of Cox-2 and PGE_2_ and induced 15-PGDH, key enzyme in PGs catabolism suggesting DHA as an anti-inflammatory and anti-invasion agent. Inhibition of Cox-2 as the therapeutic approach to breast cancer has been the focus of both clinical and laboratory investigations [45]. However, a broad inhibition of Cox-2 may result in undesirable cardiovascular side effects that are due to reduced levels of the cardio-protective prostacyclin [46, 47]. Compare with celecoxib, well known Cox-2 specific inhibitor, DHA is generally regarded as safe compounds that are well tolerated and produce few side effects. Additionally, ω3-PUFAs including DHA orchestrate several kinds of cell survival signals. For example, DHA modulates Wnt/β-catenin signal, ubiquitin/proteasomal system, autophagy, NF-κB, ROS/NRF, MMPs, VEGF and Cox-2 etc [19, 48-50]. Their effects on nontumorigenic cells have not been fully elucidated, but some studies suggest that when provided at concentrations that inhibit tumor cell growth, DHA exert little or no cytotoxic effects on normal breast cells [41, 42, 51]. Thus with further study, DHA may hold promise as nontoxic adjuvant to standard cancer therapies.

More importantly, our *in vivo* Fat-1 transgenic mice model showed a striking reduction of primary tumor growth as well as metastasis to lung suggesting that ω3-PUFAs-rich tissues provided beneficial environment to prevent both tumor progression and metastasis through inhibition of NF-κB. Even though several studies have reported that DHA could inhibit NF-κB and MMPs involving breast cancer metastasis [10, 52, 53], it has not been shown whether the suppression of MMPs/NF-κB really led the suppression of *in vivo* lung metastasis. Our study first shows the direct *in vivo* evidence that DHA can suppress lung metastasis of breast cancer in Fat-1 transgenic mice model.

Taken together, DHA showed promising effect on inhibiting tumor growth and metastasis in both *in vivo* as well as *in vitro*, these findings provide important preclinical evidence and molecular insight for the use of DHA in the chemoprevention and treatment of highly malignant human breast cancer, and substantially improve the patient's quality of life.

## MATERIALS AND METHODS

### Cell culture and reagents

MDA-MB-231 and T47D human breast cancer cell lines were purchased from the ATCC and maintained in RPMI1640 media supplemented with 10% fetal bovine serum, penicillin 100 units/ml and streptomycin 100 μg/ml at 37°C under air with 5% CO_2_. Reagents such as agarose, RPMI1640 media and fetal bovine serum were purchased from Gibco-BRL Co. DHA, EPA, AA, and PGE_2_ were purchased from Cayman Chemical (DHA and EPA were purified from algae; AA were synthesized; purity>98%). The antibodies for PARP-1, β-catenin, caspase-3, cyclin-D1, VEGF, p65, phosphor-IκBα, and IKK were purchased from Santa Cruz Biotechnology and Cox-2, 15-hydroxyprostaglandin dehydrogenase (15-PGDH) were from Cayman Chemical.

### MTT cell viability assay

Viable adherent cells were stained with MTT [3-(4,5-dimethylthiazol-2-yl)-2,3-diphenyl-tetrazolium bromide] (2 mg/ml) for 2 hrs. Media were then removed and the formazan crystals were dissolved by adding 200 μl of DMSO. Absorbance was measured at 570 nm and cell viabilities were expressed as ratios *versus* untreated control cells. All results are from independent experiments, and each experiment was performed triplicate.

### Flow cytometry

Cell cycle analysis was performed by flow cytometry. Cells were fixed with cold 70% ethanol for 30 min, and the fixed cells were centrifuged and washed twice in PBS. After centrifugation, the cell pellets were resuspended in propidium iodide (50 μg/ml) containing 0.1 μg/ml RNase A. Then the cells were incubated at 37°C for 30 min and filtered with Spectra/Mesh Nylon filter. Cell cycle distribution was analyzed by FACScan (Becton Dickinson).

### Western blot analysis

Proteins were extracted with RIPA buffer (10 mM Tris-HCl, pH8.0; 150 mM NaCl; 1% Nonidet P-40) containing protease inhibitors. Samples were resolved through a 10% SDS-polyacrylamide gel and transferred to Hybond ECL membranes (Amersham Pharmacia Biotech). Membrane was blocked in 1X TBS containing 0.1% tween 20 with 5% nonfat skim milk for 1 hr at room temperature and incubated with primary antibody for 1 hr at room temperature. After washes in TBST, the membrane was incubated with HRP-conjugated secondary antibody for 1 hr and washed three times. The membrane was visualized by an enhanced chemiluminescence method (Amersham pharmacia Biotech).

### Caspase −3 activity assay

The extracts were prepared by suspending 1×10^6^ MDA-MB-231 cells in 100 μl TTE buffer (10 mM Tris-HCl, 0.5% Triton X-100, 10 mM EDTA, pH8.0) on ice for 30 min and then centrifuged at 14,000 rpm for 10 min at 4°C. The supernatants were collected and frozen at −80°C or used immediately. Lysates (20 μl) with 10 μg of total protein were mixed with 30 μl of the enzyme reaction buffer (20% glycerol, 2 mM DTT, 20 mM HEPES, pH7.5) containing 40 μM of Ac-DEVD-AFC (a substrate for caspase-3). Caspase-3 activities were measured using a spectrofluorometric plate reader (LS-50B, Perkin-Elmer) in kinetic mode using excitation and emission wavelengths of 400-505 nm.

### Luciferase reporter assay

Cells were co-transfected with luciferase reporter plasmids (0.5 μg/well) and internal control expressing the *Renilla* luciferase. Transfections were performed using Lipofectamine (Invitrogen) according to the manufacturer's instructions. 48 hrs after transfection, the cells were rinsed twice with PBS, and cells were harvested with 200 μl of passive lysis buffer (Promega). Following a brief freeze-thaw cycle, the insoluble debris was removed by centrifugation at 4°C for 2 min at 14,000 rpm. Aliquots of the supernatant (20 μl) were then immediately processed for sequential quantitation of both firefly and *Renilla* luciferase activity (Dual-Luciferase Assay System, Promega) using a Berthold LB9507 luminometer and the activity of the *Renilla* reporter plasmid was used for normalization of transfection efficiency. All experiments were performed in triplicates.

### Immunocytochemistry

Cells seeded on glass coverslip were fixed with 4% paraformaldehyde and permeabilized with 0.5% Triton X-100 for 20 min at room temperature. Then the samples were blocked with 5% BSA for 1 h and incubated with first antibody overnight at 4°C. Samples were washed 3 times for 5 min in PBS, and then incubated with secondary antibody for 1 hr. Nuclei were counterstained with DAPI and stained cells were analyzed under a Olympus™ confocal microscope under 400× magnification.

### *In vitro* invasion and motility assay

6.5-nm polycarbonate filters of 8-μm pore size in transwell (Corning) were coated with Matrigel (Collaborative Biomedical Products). The lower compartments of transwell were filled with 500 μl of media and the Matrigel-coated filters were placed between the upper and lower compartment. The cells were plated in the upper compartments and incubated for 48 hrs. Non-invading cells on the upper surface of the filters were removed using a cotton swab. The cells that invaded to the lower surface were stained with Mayer's hematoxylin (DakoCytomation) for 1 min, and counted under a microscope. For each chamber, the number of invaded cells in 5 randomly chosen fields was counted. The motility assay was performed with the same method except for Matrigel-coated filter.

### Zymography

Conditioned media from the cell culture were analyzed for gelatin degradation activity by SDS-PAGE under non-reducing conditions. 1 mg/ml gelatin was prepolymerized on a 10% polyacrylamide gel as a substrate. Electrophoresis was carried out at 4°C. The gel was washed twice with washing buffer (50 mM Tris-HCl, pH7.5, 100 mM NaCl, 2.5% Triton X-100), followed by a brief rinsing in washing buffer without Triton X-100. Gelatinolytic activity was developed in an incubation buffer (50 mM Tris-HCl, pH7.5, 150 mM NaCl, 10 mM CaCl_2_, 0.02% NaN_3_, 1 μM ZnCl_2_) at 37°C for 48 hrs and visualized with Coomassie Blue R-250 staining.

### Immunoprecipitation and IKK kinase assay

Cell lysates were precipitated with IKK-γ antibody and protein A-sepharose beads by incubation at 4°C for overnight. The kinase assay was performed in complete kinase assay buffer (20 mM HEPES, pH7.5, 20 mM β-glycerol phosphate, 10 mM MgCl_2_, 1 mM DTT, 10 mM PNPP, 50 μM sodium vanadate, 20 μM ATP) with the addition of [γ-^32^P]ATP and 1 μg of GST-IκBα as a substrate. After 20 min at 30°C, sample buffer was added and proteins were resolved SDS-polyacrylamide gels, and phosphorylated substrates were visualized by autoradiography.

### DNA mobility shift assay (EMSA)

DNA probes used for EMSA was prepared with NF-κB binding regions; F: 5′;-GCCCAGTGGAATTCCCCAGCCT-3′; R: 5′-GCAAGGCTGGGGAATTCCACTGG-3′. End-labeled DNA probes were mixed with nuclear extracts, and reaction mixture was incubated at room temperature for 30 min and applied to a 4% non-denatured polyacrylamide gel in 0.02 M boric acid-0.05 mM EDTA. DNA-protein complex and free DNA was visualized autoradiography.

### Animals and *in vivo* tumorigenicity and metastasis

All animal-related procedures were reviewed and approved by the Institutional Animal Care and Use Committee of Chungnam National University. Fat-1 transgenic mice were kindly provided from Dr. Jing X. Kang (Department of Medicine, Massachusetts General Hospital and Harvard Medical School, Boston, MA). The Fat-1 transgenic mice (C57/BL6 genetic background) were kept under specific pathogen-free conditions in standard cages, and 8-10 weeks old female mice were used for this experiment. Syngeneic breast carcinoma cell line EO771 (1.5×10^6^ cells) was injected subcutaneous in each mouse. After inoculation, the animals were closely monitored for the development of subcutaneous tumor. The tumor's greatest dimension and the on perpendicular to it were measured every 2 to 3 days using dial calipers and expressed as 0.5×(width)^2^×length = tumor volume. For *in vivo* metastasis analysis, EO771 (1×10^6^ cells) was injected into tail vein for 4 weeks. Then the mice were sacrificed and tissues were collected for pathological examination.

### Statistical analysis

Statistical analyses were done as recommended by an independent analyst. These included the unpaired Student's test (cell viability, caspase activity, luciferase activity, tumor volume). All values were expressed as mean ± SD, and statistical significance was accepted for *p* values of <0.05.

## SUPPLEMENTARY MATERIALS FIGURES


