# Supplementary material for: Docosahexaenoic acid suppresses breast cancer cell metastasis by targeting matrix-metalloproteinases
Source: Oncotarget. 2016 Jun 23;7(31):49961–71. doi: 10.18632/oncotarget.10266 (PMC5226561; doi:10.18632/oncotarget.10266)
Supplement: Supplementary file 1 [file oncotarget-07-49961-s001.pdf]

Docosahexaenoic acid suppresses breast cancer cell metastasis by targeting matrix-metalloproteinases

SUPPLEMENTARY FIGURES

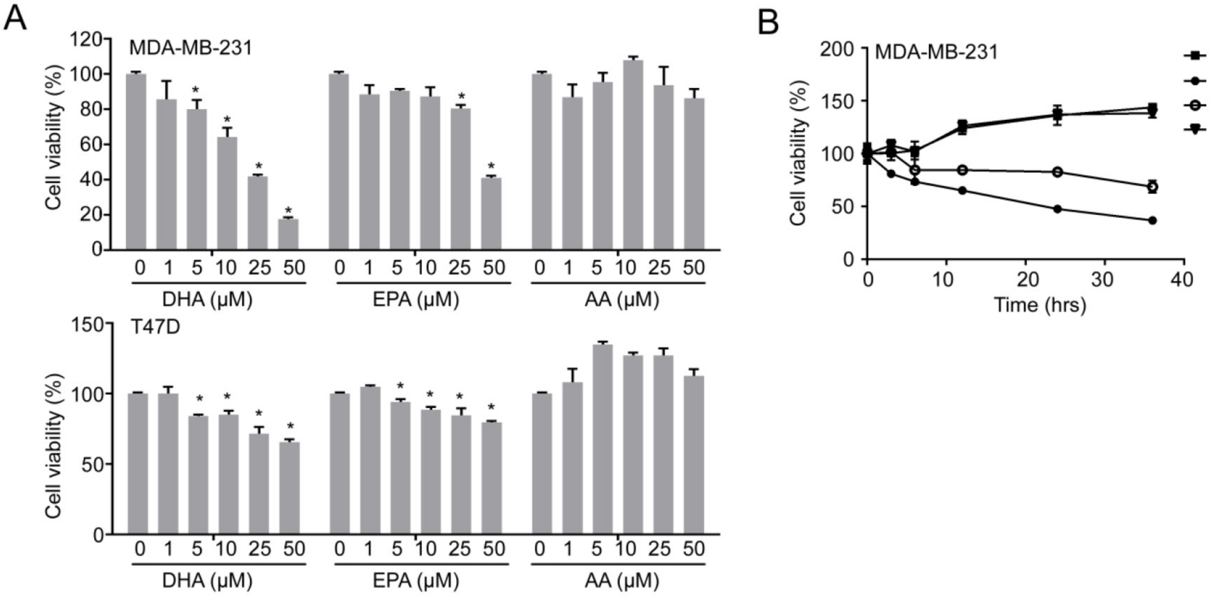

Supplementary Figure 1: DHA treatment decreases cell proliferation. A. Breast cancer cells were incubated with increasing concentration of DHA, EPA, and AA for 24 hrs, and cell growth was determined using the MTT assay. **B.** MDA-MB-231 cells were incubated with 25 µM DHA, EPA, and AA for indicated times, and cell growth was determined.

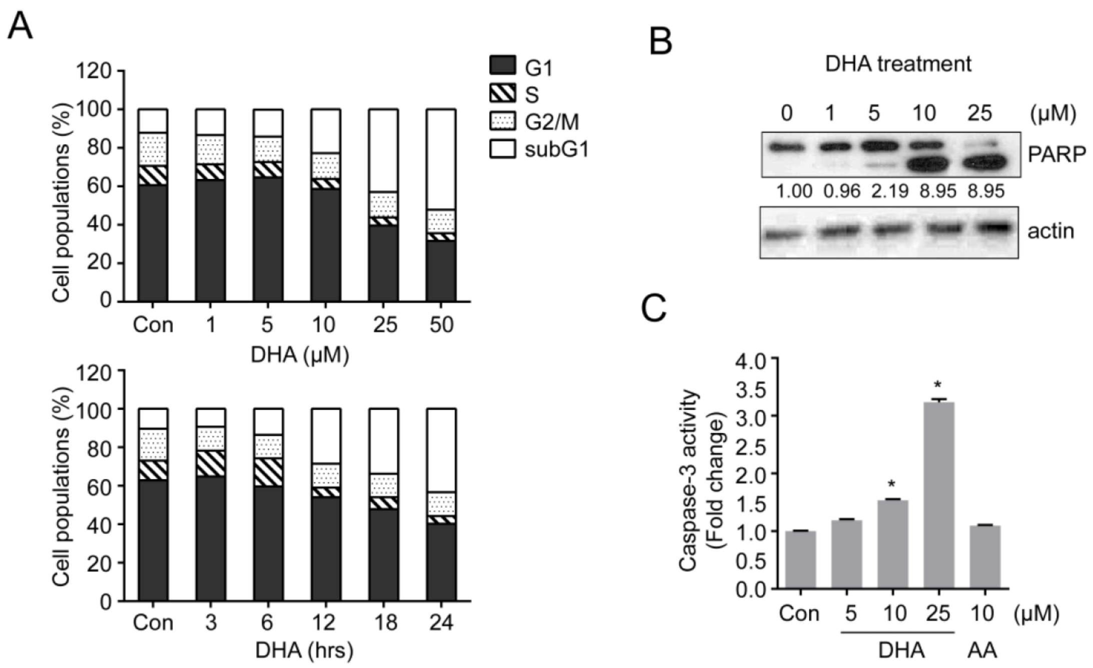

Supplementary Figure 2: DHA treatment induces apoptosis in breast cancer cell lines. A. MDA-MB-231 cells (5×10^5) were treated with different concentration (upper panel) or different duration (lower panel) of DHA. DHA fluorescence was analyzed by flow cytometry, and cell cycle distribution was calculated using Cell Quest software. **B**. MDA-MB-231 cells were treated with various concentration of DHA for 24 hrs, and PARP cleavage was examined by Western blot analysis. The densitometry ratio of cleaved PARP band intensity to actin was shown below the blot. **C.** After treatment for 24 hrs with various concentration of DHA, cells were lysed and caspase-3 activity was measured as described in Materials and Methods.

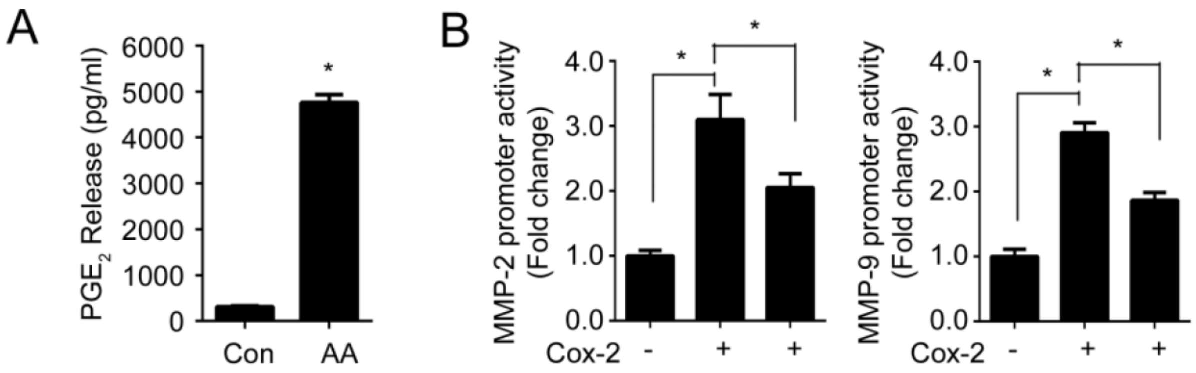

Supplementary Figure 3: AA treatment increases MMP activity by PGE_2 production. A. MDA-MB-231 cells were treated with 30 µM AA and secreted PGE_2 amount was measured by ELISA. The result is presented as the mean ± SD of three independent experiments. **B.** MDA-MB-231 cells transfected with MMP-2 or MMP-9 were pretreated with DHA (10 µM) for 2 hrs, and then Cox-2 were added. After incubation for 12 hrs, the luciferase activity was measured with dual luciferase assay.

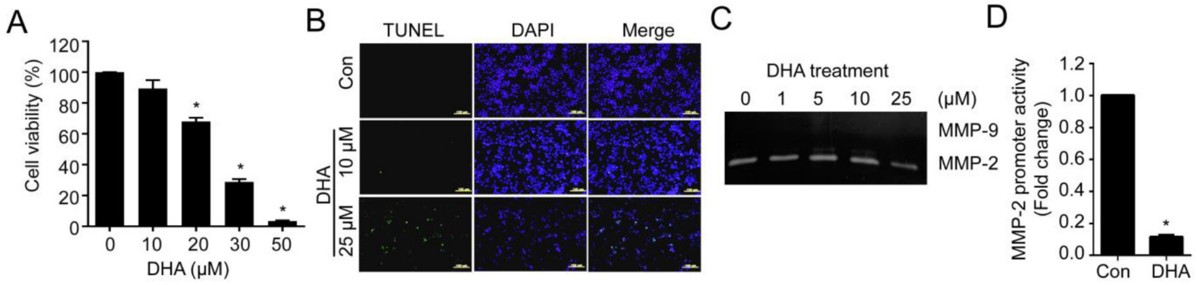

Supplementary Figure 4: DHA treatment induces apoptosis in murine breast carcinoma cell line. A. EO771, murine breast cancer cells were incubated with increasing concentration of DHA for 24 hrs, and cell viability was determined using the MTT assay. **B.** EO771 cells were treated with DHA for 24 hrs, and TUNEL assay was performed. Scale bar=100 μm. **C.** EO771 cells were treated with DHA (0, 5, 10, 25 μM) in serum-free media for 24 hrs. Then the condition media were prepared and the activity of MMP was analyzed using gelatin zymography. **D.** Cells were transfected with MMP-2 promoter, and then treated with DHA (25 μM) for 24 hrs. MMP-2 promoter activity was measured with the dual luciferase assay.
